# Moral judgment as information processing: an integrative review

**DOI:** 10.3389/fpsyg.2015.01637

**Published:** 2015-10-30

**Authors:** Steve Guglielmo

**Affiliations:** Department of Psychology, Macalester College, Saint PaulMN, USA

**Keywords:** moral judgment, blame, mental states, intuition, reasoning, emotion, information processing

## Abstract

How do humans make moral judgments about others’ behavior? This article reviews dominant models of moral judgment, organizing them within an overarching framework of information processing. This framework poses two distinct questions: (1) What input information guides moral judgments? and (2) What psychological processes generate these judgments? *Information Models* address the first question, identifying critical information elements (including causality, intentionality, and mental states) that shape moral judgments. A subclass of *Biased Information Models* holds that perceptions of these information elements are themselves driven by prior moral judgments. *Processing Models* address the second question, and existing models have focused on the relative contribution of intuitive versus deliberative processes. This review organizes existing moral judgment models within this framework and critically evaluates them on empirical and theoretical grounds; it then outlines a general integrative model grounded in information processing, and concludes with conceptual and methodological suggestions for future research. The information-processing framework provides a useful theoretical lens through which to organize extant and future work in the rapidly growing field of moral judgment.

Judging the morality of behavior is critical for a well-functioning social group. To ensure fair and effective interactions among its members, and to ultimately promote cooperation, groups and individuals must be able to identify instances of wrongdoing and flag them for subsequent correction and punishment (Boyd and Richerson, 1992; Fehr and Gächter, 2002; DeScioli and Kurzban, 2009; Tooby and Cosmides, 2010; Chudek and Henrich, 2011). Humans are quite adept at levying moral judgments and punishment upon others (Henrich et al., 2006; Boyd et al., 2010). One need only read the news on a given day to discover accusations, and appeals for punishment, of moral misconduct.

The study of morality has a rich history. Early and influential philosophers (Aristotle, 1999/330 BC) and psychologists (James, 1890/1950; Freud, 1923/1960) aimed to understand human morality and its implications for social behavior. More recent investigations have widened this scope of inquiry to examine a host of important questions concerning the evolutionary origins of morality (Hauser, 2006; Krebs, 2008), the emotional underpinnings of moral development and moral behavior (Eisenberg, 2000), the infusion of morality into everyday social interactions (Skitka et al., 2005; Wright et al., 2008), and the instantiation of moral judgment in systems of artificial intelligence (Wallach, 2010; Malle, 2014).

But an understanding of these questions requires an understanding of moral judgments themselves. Perhaps the most fundamental way in which humans categorize and understand behavior is to differentiate between *good* and *bad* (Osgood et al., 1957; Barrett, 2006b); moral judgment is an extension of this basic classification, although it is clearly more varied and complex. The literature has, for example, explored numerous related yet distinct moral judgments, including *responsibility* (Schlenker et al., 1994; Weiner, 1995), *blame* (Shaver, 1985; Alicke, 2000; Cushman, 2008; Guglielmo et al., 2009), and *wrongness* or *permissibility* (Haidt, 2001; Greene, 2007; Mikhail, 2007; Knobe, 2010).

How do humans make moral judgments? All judgments involve information processing, and although the framework of information processing has been widely implemented in models of cognitive psychology (Rosch, 1978; Marr, 1982), it has not been explicitly considered in investigations of morality. Nonetheless, existing models of moral judgment endorse such a framework, even if implicitly. With respect to moral judgment, this framework poses two fundamental questions: (1) What is the input *information* that guides people’s moral judgments? and (2) How can we characterize the psychological *processes* that generate moral judgments? Extant models of moral judgment typically examine just one of these questions, with the unfortunate result that we know little about how the questions interrelate. This article critically reviews dominant models by locating them within this guiding theoretical framework, then provides an integrative account of moral judgment and offers suggestions for future research.

## Overview of the Current Review

The study of moral judgment has grown rapidly, particularly within the past decade, yielding numerous proposed models of moral judgment. But although existing models have areas of substantial overlap, they are often studied in isolation, and empirical support for a particular aspect of a model is often taken as evidence for the veracity of the model as a whole. Further, these models investigate a suite of different moral judgments—including responsibility, blame, and wrongness, among others—that share commonalities but are not identical. A comprehensive and systematic analysis of moral judgment that assesses existing models in their entirety and in relationship to other models is sorely needed. Such an analysis would enable clarification of the claims of and support for existing models, explication of their areas of agreement and divergence, and organization within a unifying theoretical framework. This article provides such an analysis, critically evaluating existing models on empirical and theoretical grounds while also locating them within an overarching framework that emphasizes the information processing nature of moral judgment.

Existing models of moral judgment can be organized around their two fundamental goals. The first goal is to account for the particular information content that underlies people’s moral judgments: the aspects of a behavior, or the agent who performed it, that lead people to hold the agent responsible, blameworthy, and so on. Models that focus on this goal are here referred to as *information models* (Shaver, 1985; Schlenker et al., 1994; Weiner, 1995; Cushman, 2008). These models include a subclass of *biased information models* (Alicke, 2000; Knobe, 2010), which hold that the very perceptions of such information content are driven by prior moral judgments. The second goal is to identify the psychological processes that generate moral judgments, including the extent to which these judgments are driven by intuitive or emotional processes on the one hand, or by deliberative processes on the other. Models that focus on this goal are here referred to as *processing models* (Haidt, 2001; Greene, 2007).

The goals of information models and processing models can be regarded as largely independent of one another. Revealing the importance of particular information features does not thereby establish the relative importance of intuitive or deliberative processes; similarly, revealing the importance of these processes does not thereby establish which information content drives moral judgments (cf. Rosch, 1978). A metaphor helps illustrate the distinction: we can separately examine the directions of travel on the one hand (information models) and the modes of travel on the other (processing models), although we will clearly be most successful by examining them together.

In reviewing the most prominent models within each of these classes, this article has three general aims: specifying the claims of each model; clarifying how the models compare to one another; and evaluating each model on empirical and theoretical grounds. The article then outlines a general information-processing view of moral judgment and highlights a specific recent model that adopts an information-processing approach (Malle et al., 2012, 2014). Finally, the paper offers conceptual and methodological suggestions for future research.

Before proceeding, though, we must first establish the domains in which moral judgment is relevant. Which general kinds of behavior have the capacity to elicit moral judgments? *Harm* and *fairness* are paradigmatic domains of moral judgment (Kohlberg, 1969; Turiel, 1983), but recent work has demonstrated the additional importance of *loyalty*, *authority*, and *purity* domains (Haidt, 2007, 2008; Graham et al., 2009, 2011; Haidt and Graham, 2009). Some scholars have argued, in contrast, that harm represents the single superordinate moral domain (Gray et al., 2012), and others suggest that moral judgments fundamentally reflect concerns about maintaining social relationships (Rai and Fiske, 2011). Despite the promise of a multitude of perspectives, extant research on moral judgment has been dominated by investigations of harm and fairness, which will therefore, by necessity, be the primary focus of the current analysis.

## Information Models

Information models specify the features of an agent’s behavior that shape people’s moral judgments. Early models emphasized the concept of responsibility (Shaver, 1985; Schlenker et al., 1994; Weiner, 1995) and although they have provided noteworthy contributions, the concept of responsibility has proven to be incomplete in capturing the sensitivity of people’s moral judgments, as we will see. More recent models, reviewed subsequently, have examined less ambiguous types of moral judgments such as wrongness or blame (Cushman, 2008).

### Models of Responsibility

#### Shaver: Responsibility and Blame

Building upon the seminal work of Heider (1958), Shaver (1985) offers one of the earliest comprehensive psychological accounts of the particular components that underlie moral judgment. Shaver differentiates between *responsibility* and *blame* judgments, asserting that the latter presuppose the former. The heart of the model concerns responsibility judgments, which Shaver (1985, 1996; Shaver and Drown, 1986) argues are guided by five elements: the agent’s *causal* contribution; *awareness* of negative consequences; *intent* to cause the event; degree of *volition* (e.g., freedom from coercion); and appreciation of the action’s *wrongness*. Indeed, moral evaluations are sensitive to an agent’s causal and intentional involvement in a negative action (Darley and Shultz, 1990; Ohtsubo, 2007; Lagnado and Channon, 2008), differentiate between responsibility and blame (Harvey and Rule, 1978), and follow a causality → responsibility → punishment pattern in particular (Shultz et al., 1981).

However, some aspects of the model are puzzling. Shaver (1996, p. 246) suggests that in some cases full responsibility applies yet blame is nullified—namely, when an agent has acceptable *justifications*, which “claim a larger positive social goal for which the intentional harm was produced,” or *excuses*, which “claim that the particular consequences were not intended.” But justifications seemingly appeal to Shaver’s *wrongness* element of responsibility, and excuses seemingly appeal to the *intentionality* element. Thus, justifications and excuses should also weaken responsibility, not just blame. Further, Shaver claims that blame is assigned “after the perceiver *assesses and does not accept*” the offender’s justifications and excuses (Shaver and Drown, 1986, p. 701, emphasis added). Although justifications and excuses can moderate blame substantially—socially desirable reasons or motives mitigate blame (Lewis et al., 2012; Piazza et al., 2013), whereas socially undesirable reasons or motives exacerbate blame (Reeder et al., 2002; Woolfolk et al., 2006)—there is no evidence that perceivers necessarily consider these factors prior to assessing blame. The emphasis on withholding blame until evaluating justifications and excuses may be ideal for a prescriptive model of how people *should* assign responsibility and blame but not for a descriptive model of how people actually make these judgments. As it turns out, Shaver’s (1985) model is intended to be prescriptive; thus, its explanatory aim differs notably from descriptive models of moral judgment, on which the remainder of this paper will focus.

#### Weiner: Responsibility and Social Conduct

Weiner (1995) examines two related phenomena: people’s judgments of responsibility and their emotional and behavioral reactions to others’ behavior. In this model, considerations of controllability drive people’s responsibility judgments, which in turn guide their emotional responses (e.g., anger vs. sympathy) and social actions (e.g., retaliation vs. helping) toward others. Weiner, like Shaver, holds that causality is a necessary but not a sufficient condition of responsibility: “the cause must be controllable if the person is to be held responsible” (Weiner, 1995, p. 11). If the cause of a negative outcome is “uncontrollable”—such as a heart attack or a low mental aptitude—responsibility judgments are withheld. Weiner (1995) reviewed a wealth of evidence showing that perceptions of controllability influence people’s judgments of responsibility.

Although Weiner (1995) identifies several critical inputs to moral judgment, the model omits one key factor: intentionality. The distinction between intentional and unintentional actions is critical for moral judgment (Darley and Shultz, 1990; Ohtsubo, 2007; Gray and Wegner, 2008; Lagnado and Channon, 2008), but the concept of controllability is too broad to capture this distinction. On Weiner’s (1995) model, both intentional and unintentional behaviors will often be “controllable,” because the agent could have acted differently. But people’s moral judgments distinguish between intentional behavior and negligent behavior, even if the negative consequences are identical (Cushman, 2008), which is reflected in the legal distinction between (intentional) murder and (unintentional) manslaughter. While Weiner’s model cannot readily distinguish between intentional and unintentional behavior generally, the notion of controllability (i.e., consideration of the agent’s capacity to foresee and prevent the negative outcome) nonetheless succeeds in explaining moral judgments about unintentional behavior specifically.

#### Schlenker et al.: Triangle Model of Responsibility

Schlenker et al. (1994) propose that responsibility judgments are shaped by the links between a prescription, an event, and an agent’s identity. In particular, “people are held responsible to the extent that a clear, well-defined set of prescriptions is applicable to the event (prescription-event link), the actor is perceived to be bound by the prescriptions by virtue of his or her identity (prescription-identity link), and the actor seems to have (or to have had) personal control over the event, such as by intentionally producing the consequences (identity-event link)” (p. 649). The first link resembles Shaver’s wrongness element and the third resembles Weiner’s controllability element; the second link (prescription-identity) identifies the importance of an agent’s obligations in the given situation. Schlenker et al. (1994) provided evidence that each link independently contributed to people’s judgments of how responsible a worker was for his or her job performance. However, Schlenker et al.’s (1994) model has the same critical weakness as Weiner’s: it omits intentionality.^1^ As discussed above, the concept of controllability is too coarse to capture the distinction between intentional and unintentional behavior; although both types of behaviors typically are “controllable,” people’s moral judgments differentiate markedly between them.

#### Limitations of Responsibility Models

Extant models of responsibility highlight several components that shape people’s moral judgments, including causality, controllability, and obligation. But these models fall short as comprehensive accounts of moral judgments due to their prescriptive emphasis (Shaver, 1985) or their omission of intentionality (Schlenker et al., 1994; Weiner, 1995). A further concern is that the concept of responsibility itself has taken on a host of meanings in the literature and is therefore not an ideal candidate for understanding moral judgment. Responsibility sometimes indicates mere causality—for example, Harvey and Rule (1978) examined “whether moral evaluations and causal responsibility are distinct judgmental dimensions,” and Critchlow (1985) found that responsibility and causality judgments were similar across a range of behaviors. It can also denote general obligations (e.g., “Who is responsible for cleaning up?”), or it can simply be synonymous with blame (e.g., “Moral responsibility refers to the extent to which the protagonist is worthy of blame”; Shultz et al., 1981, p. 242, emphasis in original). Consequently, responsibility either lacks clear moral content (e.g., when it stands for causality) or is redundant with less ambiguous moral judgments (e.g., blame). Recent models have therefore examined less equivocal moral judgments while nonetheless incorporating key insights from early responsibility models.

### Cushman: Causal-intentional Model of Wrongness and Blame

**FIGURE 1:**
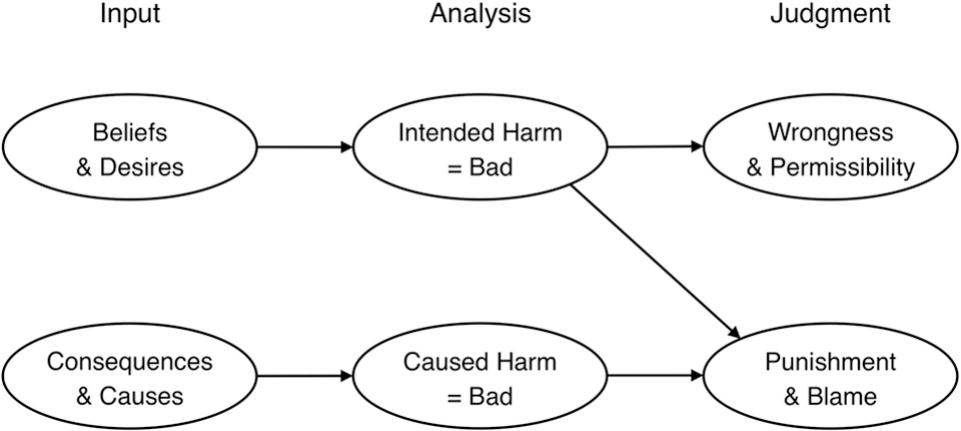
**Cushman’s causal-intentional model of moral judgment**. Reprinted from Cushman (2008) with permission from Elsevier.

Cushman’s (2008) causal-intentional model aims to account for distinct moral judgments of wrongness and blame. The model (Cushman, 2008, p. 364), shown in **Figure 1**, asserts that information about mental states underlies wrongness judgments, whereas mental states and consequences/causality jointly underlie blame judgments. More specifically, Cushman argues that inferences about *beliefs* (whether the agent believed his behavior would cause harm) and *desire*s (whether the agent wanted to cause harm) independently contribute to judgments of both wrongness and blame. The joint presence of these two mental states typically connotes that the behavior in question was intentional (Malle and Knobe, 1997a), and whereas many studies on moral judgment manipulate intentionality, Cushman (2008) examines beliefs and desires independently. In addition to these mental states, Cushman’s (2008) model holds that *causes and consequences* (i.e., what actually happens as a result of an agent’s action) influence blame. Agents may or may not actually cause harm—regardless of their intent—and blame will track the amount of harm (e.g., as in the differential punishment assigned to actual vs. attempted murder, both of which imply an intention to harm but differ in whether the harm actually occurred).

#### Evidence for Cushman’s Causal-intentional Model

The importance of causality and intentionality in moral judgment is well established. Blame is greater to the extent that an agent is seen as the cause of a negative event (Lagnado and Channon, 2008), and a substantial body of evidence shows that intentional negative actions are blamed and punished more than unintentional negative actions (Darley and Shultz, 1990; Ohtsubo, 2007; Gray et al., 2012). Further, culpable beliefs, desires, and motives increase blame both among adults (Young and Saxe, 2009; Tannenbaum et al., 2011; Inbar et al., 2012) and among children (Suls and Kalle, 1978; Nelson-Le Gall, 1985; Zelazo et al., 1996).

Cushman (2008) tested the model’s more specific claims by independently varying belief, desire, and negative consequences, and then probing wrongness and blame judgments. For example, one vignette described Jenny, who was working in a sculpture class with a partner. Jenny did [not] want to burn her partner (desire present [absent]) and did [not] think that welding a piece of metal would burn her partner (belief present [absent]); Jenny welded the metal, which did [not] burn her partner (consequence present [absent]). Wrongness judgments were influenced by beliefs and desires but were essentially unaffected by consequences. Blame judgments told a different story: mental states continued to be critical, but blame was additionally influenced by consequences. Together, the results suggest that whereas wrongness is guided solely by mental states, blame is guided by mental states and consequences.

Other evidence for Cushman’s (2008) model comes from studies on “outcome bias” or “moral luck,” the pattern whereby an agent receives more blame for a behavior that *happens to have* bad consequences than for one that does not (or for a behavior whose bad consequences are worse than another’s). For example, someone who carelessly backs out of a parking spot would receive more blame if he happened to hit a bystander than if he happened not to. A great deal of evidence reveals such outcome bias effects. For example, Mazzocco et al. (2004) found that while blame was mostly predicted by mental states (negligence, in their studies), it was partially predicted by negative outcomes too. Cushman et al. (2009) showed that punishment of a person’s act of rolling a die (which could produce negative, positive, or neutral outcomes) was higher not only when the person intended to cause a bad outcome but also when a bad outcome occurred by chance. These patterns are consistent with Cushman’s model, showing that blame is jointly a function of mental states and, to a lesser extent, consequences.

#### Limitations of Cushman’s Model

Cushman highlights the role of consequences and causality, which indeed shape blame. But whereas the model treats these as identical inputs to blame (see **Figure 1**), in truth they refer to very different features. Consequences denote whether a negative outcome in fact occurred; causality denotes whether the agent in question was the cause of this outcome. The distinct roles of these two factors can be illustrated by a pattern in Cushman’s (2008) Study 3, which showed that people gave an agent more blame when the agent caused harm than when the harm was caused by someone else. In both cases, the negative consequences were identical; what differed was whether the agent was the cause of the consequences or not. This suggests that an agent’s causal role in producing harmful consequences is more important for blame than merely whether such consequences occurred.

One possibility not directly addressed by Cushman’s model is that causal and intentional factors influence one another. Appearing to contradict this possibility is Cushman’s finding that mental states and consequences had no interaction effect on moral judgments. But Cushman’s vignettes *manipulated* mental state and consequence information, making obvious the presence or absence of each one. In contrast, people usually need to make these inferences themselves, and information about one factor will often guide inferences about another. For example, if a person performs an action that she believes will cause harm, people will tend to infer that she wanted to bring about harm (Reeder and Brewer, 1979; Guglielmo and Malle, 2010; Laurent et al., 2015a). Moreover, if an agent causes a negative outcome, people may infer corresponding culpable mental states (Pettit and Knobe, 2009; Young et al., 2010).

### Summary of Information Models

Information models seek to characterize the critical information elements that guide people’s moral judgments. Extant models have examined a range of different moral judgments, and have identified key distinctions between them. Yet several important consistencies have emerged. Moral judgments stem from identifying the occurrence of a negative event and the causal involvement of an agent. Moreover, perceivers consider whether the agent acted intentionally, as well as the agent’s more specific mental states such as desires (including reasons and motives) and beliefs (including foresight and controllability). Notably, the importance of these features emerges early in development. Six-month olds generally dislike those who cause harm (Hamlin et al., 2007) and 8-month olds are sensitive to intentions, preferring an agent who intends to help over one who intends to harm (Hamlin, 2013). Further, 8-month olds prefer that agents respond with harmful behavior to an antisocial other (Hamlin et al., 2011), illustrating a rudimentary understanding that certain reasons or motives may permit an otherwise disfavored negative act. Lastly, children are sensitive to an agent’s beliefs and the controllability of behavior, viewing negligent harm as morally worse than purely accidental harm (Darley and Shultz, 1990) and freely chosen harm as worse than behaviorally constrained harm (Josephs et al., 2015).

## Biased Information Models

Biased^2^ information models hold that although the critical information elements identified by the preceding models—causality, intentionality, and other mental states—may shape explicit moral judgments such as blame, these elements are themselves directly influenced by more implicit moral judgments about the badness of an outcome or an agent (Alicke, 2000; Knobe, 2010). These models reverse the order of judgment postulated by information models in suggesting that moral judgments can precede, rather than just result from, causal and mental analysis. Although biased information models are not strictly incompatible with the preceding information models (since neither type explicitly denies the existence of the processing order favored by the other type), these two types clearly disagree about which processing order is most prevalent and thus has the most explanatory power with respect to people’s moral judgments.

### Alicke: Culpable Control Model of Blame

Alicke’s (2000) culpable control model specifies the impact of “spontaneous evaluations” on causal and mental judgments (Alicke refers to these judgments as “structural linkage assessments”), as well as on blame. Spontaneous evaluations are affective reactions that arise “in response to information concerning a person’s intentions, behaviors, or the consequences they produce” (Alicke, 2000, p. 558). Structural linkage assessments refer to judgments about mental states (e.g., intentions and foresight) and causality, which are of course the key elements identified by information models. Alicke (2000, p. 559, emphasis added) holds that “spontaneous evaluations influence blame attributions both *directly* as well as *indirectly* by means of their effect on more deliberate structural linkage assessments”. To facilitate comparison to other models, “structural linkage assessments” are hereafter called “causal-mental judgments.”

#### Clarifying the Predictions of Alicke’s Model

**FIGURE 2:**
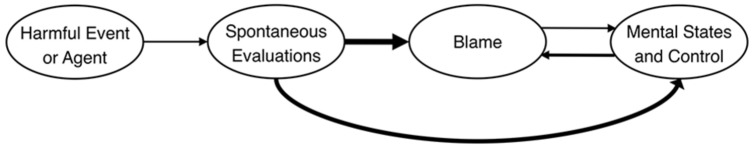
**Implied graphical representation of Alicke’s culpable control model**.

Although Alicke does not provide a full graphical depiction of his model, we can construct one from his discussion (Alicke, 2000, pp. 564–568) of five unique combinations between spontaneous evaluations, blame, and causal-mental judgments (e.g., spontaneous evaluations may directly influence blame, but blame may have no further influence on causal-mental judgments, etc.). Three combinations posit direct effects of spontaneous evaluations on blame; one posits an indirect effect (via causal-mental judgments); one posits simultaneous direct and indirect effects. **Figure 2** combines these into a single representation of Alicke’s model, whereby the more proposed pathways between pairs of variables (Alicke, 2000, p. 565), the thicker the arrow connecting them. From this construction, we see that the spontaneous evaluations → blame link is the strongest, followed by the spontaneous evaluations → causal-mental judgments link, and the causal-mental judgments → blame link (the last two of which constitute the indirect effect of spontaneous evaluations on blame). In short, Alicke’s model implies that the direct effect of spontaneous evaluations on blame is much larger than the indirect effect.

Once represented explicitly in this way, we see that the model contains every pairwise connection between spontaneous evaluations, blame, and causal-mental judgments. This quality—that the model is “saturated”—makes model evaluation difficult, as saturated models accommodate every relationship and therefore cannot by falsified on statistical grounds.^3^ To evaluate Alicke’s model fairly, either the direct or the indirect effect of spontaneous evaluations on blame should be omitted, thereby avoiding the problem of model saturation. Emphasizing the direct effect is consistent with the graphical implication of Alicke’s model (**Figure 2**) and with the claim that perceivers “search selectively for information that supports a desired blame attribution” (Alicke, 2000, p. 568). In other words, blame precedes and motivates assessments of mental states and causality. Consequently, the strongest evidence for the model will be evidence of a direct effect of spontaneous evaluations on blame; to the extent that the relationship is *indirect* (via causal-mental judgments), this should not be taken as support for blame validation.

#### Evidence for Alicke’s Culpable Control Model

##### Direct effect

Alicke’s (2000) section on Direct Spontaneous Evaluation Effects reviewed a single study that found an effect of outcome negativity on blame that was not mediated by causality ratings (Alicke et al., 1994). It is not clear, though, whether Alicke et al. (1994) assessed ratings of causality, nor are mediation analyses reported. Mazzocco et al. (2004) provide one of the few investigations of the mediational model implied by Alicke’s model. In their studies, a protagonist killed an intruder who turned out to be either his daughter’s boyfriend or a dangerous criminal. The critical prediction for Alicke’s (2000) model is that the direct effect (the outcome → blame path, after accounting for the effect of negligence on blame) should be stronger than the indirect effect (the negligence → blame path, after accounting for the effect of outcome on negligence). However, the results showed the reverse: the indirect effect was significant in all four studies (average r = 0.42), whereas the direct effect was significant in just one study (average r = 0.17).^4^

Alicke and Zell (2009) examined whether a protagonist’s likeability—a possible measure of spontaneous evaluations—influenced blame. In one study, a socially likeable agent (who was polite to a policeman; volunteered at a homeless shelter) or unlikeable agent (who was rude to a policeman; lied to his boss) accidentally punched and injured an innocent woman. Blame was higher for the unlikeable character than the likeable one, and this effect was mediated by likeability ratings.

##### Indirect effect

As we have seen, there is little existing evidence for a direct effect of spontaneous evaluations on blame, which is the primary prediction of Alicke’s model. We can nonetheless consider the evidence for an indirect effect; if such an effect stems from a motivational bias, whereby people “want” to perceive greater negligence (or causality, etc.), then this pattern may support Alicke’s model.

Alicke (1992) found that an unlikeable agent (who was trying to hide cocaine) was judged more causally responsible for his ensuing car accident than was a likeable agent (who was trying to hide a gift). Participants in Mazzocco et al.’s (2004) studies saw an agent as more negligent when his actions had more negative consequences (e.g., the intruder he killed was his daughter’s boyfriend vs. a criminal). Similarly, Alicke et al. (1994) found higher ratings of negligence and irresponsibility in the boyfriend vs. criminal condition. In all cases, the claim of Alicke’s model is that spontaneous evaluations—triggered by the negativity of the agent and/or the outcome in question—led to enhanced ratings of causality and negligence, which thereby enhance blame.

#### Limitations of Alicke’s Model

The major challenge to Alicke’s model is that its primary claim of a direct effect of spontaneous evaluations on blame is not robustly supported. In a possible exception, Alicke and Zell (2009) showed that likeability predicted blame, but the assessed ratings of causality were not included in the mediation models, leaving it unclear whether likability influenced blame directly or indirectly (via causality). Further, the authors asked about the agent’s “blameworthiness” in general (rather than for the specific act of injuring the woman), making it possible that the unlikeable agent received greater blame as a result of performing additional negative actions (e.g., being rude, lying).

Evidence consistently shows that the indirect effect from negative outcomes to blame—mediated by causal-mental judgments—is stronger than the direct effect. Could this indirect effect constitute an undue motivational bias? Alicke (2000, p. 566) indeed argues that, “Although a victim’s loathsome character is irrelevant for determining legal responsibility (Federal Rules of Evidence, 2009, Rule 404b), there is little doubt that a jury’s sympathies and antipathies for the victim influence their verdicts.” Interestingly though, while Rule 404b forbids character evidence from informing guilt directly, it *does* permit such evidence to guide mental-state inferences: “Evidence of other crimes, wrongs, or acts…may, however, be admissible for other purposes, such as proof of motive, opportunity, intent, preparation, plan, knowledge, identity, or absence of mistake or accident.”

This legally permissible pattern of influence may explain how negative character or outcome information, which is generally more diagnostic than positive information (Reeder and Brewer, 1979; Skowronski and Carlston, 1987, 1989), shapes causal-mental judgments. People might (reasonably) infer that the drug-hiding agent in Alicke’s (1992) car accident story was more reckless, impulsive, or apathetic than the gift-hiding agent, and these inferences may help account for the discrepancy in assessments of causality. Similarly, negative outcomes often bring to bear other inferences about foresight or preventability. When an agent mistakenly kills his daughter’s boyfriend (as in Alicke et al., 1994), participants might infer that the agent could or should have known about the boyfriend’s presence, thus blaming the agent for his unwarranted false belief that the intruder was a criminal. Consistent with this interpretation, Young et al. (2010) showed that people assign substantial blame to agents who act upon false beliefs, regardless of whether they ultimately caused harm. The influence of negative outcome or character information on causal-mental judgments is therefore likely *informational*, not *motivational*, since negative information is a diagnostic indicator of related inferences about dispositions, foresight, and preventability (cf. Uttich and Lombrozo, 2010).

Alicke’s model nonetheless raises the important possibility that early affective responses may impact later phases of moral judgment. Future research must be careful to determine whether this link is affective/motivational or informational in nature. If it turns out to be the former, then information models of moral judgment will need to specify how early evaluative responses shape later judgments (e.g., causal-mental judgments and blame).

### Knobe: Moral Pervasiveness Model

**FIGURE 3:**
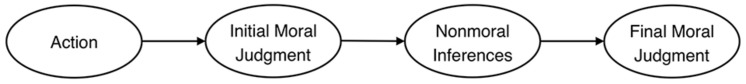
**Knobe’s moral pervasiveness model**.

Knobe’s moral pervasiveness model (Pettit and Knobe, 2009; Knobe, 2010) depicted in **Figure 3** (adapted from Phillips and Knobe, 2009) asserts that “initial moral judgments” influence causal-mental judgments. Knobe’s (2003a,b) earliest work suggested that initial moral judgments were judgments of blame; more recent specifications view them as akin to judgments of goodness or badness: “people’s judgments of good and bad are actually playing a role in the fundamental competencies underlying their concept of intentional action.” (Knobe, 2006, p. 221). Knobe’s model posits that initial moral judgments influence *all* the components identified by information models, including intentionality (as well as desires, beliefs, or decisions; Knobe, 2010), causality (Knobe and Fraser, 2008) and reasons for acting (Knobe, 2007).

Whereas Alicke’s account is that rudimentary initial moral judgments *can* guide causal-mental inferences, Knobe’s account is that the very concepts underlying these inferences are fundamentally shaped by moral concerns: “Moral judgment is pervasive; playing a role in the application of *every* concept that involves holding or displaying a positive attitude toward an outcome” (Pettit and Knobe, 2009, p. 593). Thus, both models posit that people have immediate evaluative reactions, which then influence their causal-mental assessments. Alicke holds that this is a *motivational* process of blame-validation, whereby people exaggerate their causal-mental judgments to justify their initial negative evaluations. In contrast, Knobe holds that these effects reflect a *conceptual* influence—by virtue of viewing an action as bad, people directly perceive more culpable causal-mental features.

#### Evidence for Knobe’s Moral Pervasiveness Model

Knobe’s model is supported by previously reviewed evidence for (indirect) effects of negativity on blame that are mediated by causal-mental judgments. For example, Mazzocco et al. (2004) showed a strong outcome → blame effect that was mediated by negligence judgments, consistent with Knobe’s claim that negativity enhances culpable mental state judgments (and, thereby, blame).

The most widely known evidence for Knobe’s model comes from the “side-effect effect” (Leslie et al., 2006), whereby people view negative side effects as more intentional than positive ones. In the original demonstration of the effect (Knobe, 2003a), a CEO adopted a program that increased profits, with a side effect of harming [helping] the environment. The CEO stated, “I don’t care at all about harming [helping] the environment,” thereby putatively indicating a lack of desire for the side effect. Most people said that harming the environment was intentional but helping was unintentional, a pattern that has emerged across variations in age and vignette content (Leslie et al., 2006; Cushman and Mele, 2008; Mallon, 2008).

Other evidence shows that morality appears to impact a host of other non-moral judgments. People more often judged that the harming CEO, as compared to the helping CEO, *intended* the outcome (Knobe, 2004; McCann, 2005), *knew* about the outcome (Beebe and Buckwalter, 2010), *decided to* bring about the outcome, and was *in favor of* the outcome (Pettit and Knobe, 2009). Moral judgments also appear to influence assessments of causality (Knobe and Fraser, 2008) and freedom (Phillips and Knobe, 2009) in a similar fashion.

#### Limitations of Knobe’s Model

One challenge to Knobe’s account is that the harming and helping CEO scenarios differ not only in moral valence but also in the agent’s implied attitude toward that outcome. Since people expect others to prevent negative events and foster positive ones, professed indifference about an outcome constitutes evidence of a welcoming attitude when the outcome is negative but not when it is positive. Adjusting these mismatched attitudes by making the harming CEO less welcoming and the helping CEO more welcoming led people to judge the two actions as equally intentional (Guglielmo and Malle, 2010). Moreover, people rarely said the side effect was intentional once given other options of describing the situation; they instead indicated that the CEO knowingly brought about the outcome, and this pattern was identical for the harming and helping scenarios (Guglielmo and Malle, 2010; Laurent et al., 2015b). These findings challenge the claim that moral judgments impact the “fundamental competencies underlying [people’s] concept of intentional action” (Knobe, 2006, p. 221).

Knobe’s model does not specify what initial moral judgments actually are and therefore what triggers them. The model requires that these judgments are *not* shaped by causal-mental inferences, since such inferences are themselves posited to be guided by initial moral judgments. By virtue of what, then, do initial moral judgments arise? The clearest possibility is that these “judgments of good and bad” are driven by outcomes or consequences. However, several experimental variations reveal low intentionality ratings despite the presence of a bad outcome, such as when the agent “felt terrible” about the outcome (Phelan and Sarkissian, 2008; see also Cushman and Mele, 2008). Even the paradigmatic side-effect effect requires not just the occurrence of a negative outcome but also the agent’s *knowledge* that it will occur; when this knowledge is absent, people rarely judge the outcome intentional (Nadelhoffer, 2006; Pellizzoni et al., 2010). Thus, the initial moral judgments of Knobe’s model are sensitive at least to the agent’s knowledge and attitude (Guglielmo, 2010), challenging the claim that such moral judgments occur prior to and without consideration of an agent’s mental states.

A final challenge is that many of the moral pervasiveness patterns likewise emerge for non-moral norm violations. This is because breaking a norm (moral or otherwise) provides diagnostic evidence of the agent’s desires, intentions, and causal role (Jones and Davis, 1965; Harman, 1976; Machery, 2008). For example, people judged it more intentional to break rather than conform to a dress code (Guglielmo and Malle, 2010), or to make unconventionally rather than conventionally colored toys (Uttich and Lombrozo, 2010). Puzzlingly, Knobe has sometimes emphasized norm violation in general (Knobe, 2007; Hitchcock and Knobe, 2009), and other times *moral* violation in particular (Pettit and Knobe, 2009; Knobe, 2010). In one striking study that pitted norm violation against morality (Knobe, 2007), the side effect of the CEO’s action was either violation of a Nazi law (a good but norm-violating outcome) or conformity to the law (a bad but norm-conforming outcome). People viewed the norm-violating (but good) outcome as intentional far more often (81%) than the norm-conforming (but bad) outcome (30%), demonstrating the supremacy of norm violation over moral concerns.

### Summary of Biased Information Models

Biased information models raise the intriguing possibility that causal-mental assessments—which are typically viewed as inputs to moral judgment—are themselves driven by more basic moral judgments. However, the current analysis suggests that this fundamental claim of biased information models is not, at present, well supported. For one, these models have not empirically assessed the operative early moral judgments. Moreover, although negativity impacts non-moral assessments, this pattern can often be accounted for without appealing to motivational or conceptual influences. Norm-violating information provides grounds for related diagnostic inferences. Consequently, the patterns predicted by biased information models emerge even for non-moral norm violations, and the patterns for moral violations become far weaker when controlling for the relevant diagnostic information.

## Processing Models

The models reviewed so far are concerned primarily with the information components that underlie moral judgments. A distinct set of models—here called processing models—has a different emphasis, instead focusing on the psychological processes that are recruited when people determine whether a behavior is immoral or worthy of blame. Although many possible forms of processing might be examined, the literature has typically examined two putatively competing types: intuitive or emotional processes on the one hand, and deliberative or reason-based processes on the other.

### Haidt: Social Intuitionist Model of Moral Judgment

**FIGURE 4:**
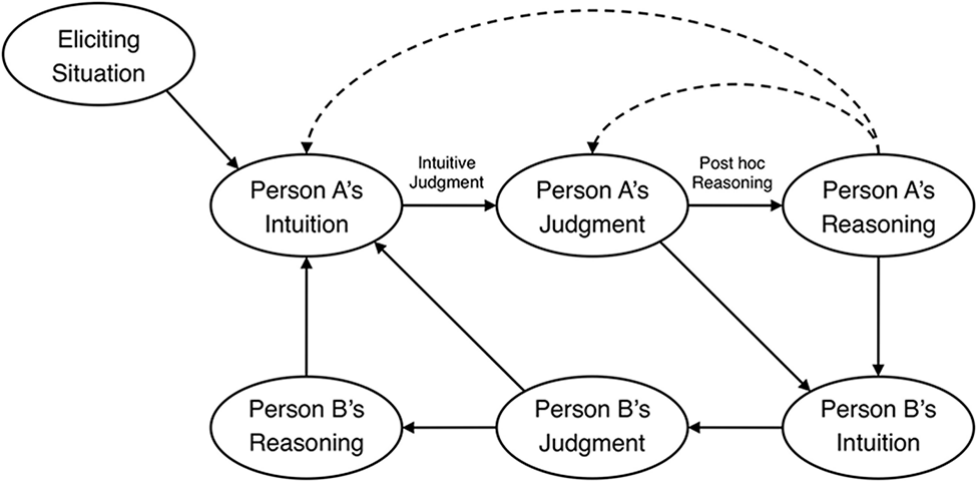
**Haidt’s Social Intuitionist Model of moral judgment**. Reprinted from Haidt (2001) with permission from APA

Haidt’s (2001, p. 815) Social Intuitionist Model, shown in **Figure 4**, asserts that “moral judgment is caused by quick moral intuitions and is followed (when needed) by slow, ex post facto moral reasoning” (p. 817). This statement contains two distinct claims about the intuitive nature of moral judgment. One is a “negative” claim that reasoning usually does not precede, but rather follows from, moral judgment. This claim, shown in **Figure 4** as the *post hoc reasoning* link, challenges the long tradition of reason-based moral judgment models (Kant, 1785/1959; Piaget, 1932/1965; Kohlberg, 1969; Turiel, 1983). The second, “positive,” claim is that intuitions or emotional responses directly cause moral judgments (the *intuitive judgment* link).

The *eliciting situation* element of Haidt’s model denotes the kinds of situations that are apt to generate moral intuitions and, therefore, moral judgments. Recent research on these “taste buds” of morality (Haidt and Joseph, 2007) suggests that there are five broad moral domains: *harm*, *fairness*, *ingroup*, *authority*, and *purity* (Graham et al., 2009; Haidt and Graham, 2009; Haidt and Kesebir, 2010). It remains to be seen whether the fundamental links in Haidt’s model between intuition, judgment, and reasoning are true for each of these five moral domains; most evidence for the model, as we will see, comes from studies examining purity.

Close inspection reveals that Haidt emphasizes a different type of moral judgment than that examined by information models. Information models assume or stipulate that the moral judgment process begins with the identification of a negative event (e.g., a particular harmful outcome), and thus causal-mental judgments are relevant only insofar as they tie an agent to the event. In contrast, Haidt’s model arguably assesses how people determine what constitutes a negative event in the first place. Studies of Haidt’s model always hold constant the agent’s causal and intentional involvement, so observed differences in moral judgments can be ascribed not to these factors but to whether perceivers viewed the behaviors as negative.

#### Evidence for Haidt’s Social Intuitionist Model

Haidt’s (2001) model can be supported by two distinct lines of evidence: one corresponding to the *post hoc* reasoning claim that moral reasoning follows moral judgment, and one to the intuitive judgment claim that intuitive or emotional responses directly guide moral judgments.

##### Post hoc reasoning

Reasoning processes are sometimes deployed to obtain confirmation for favored conclusions, rather than to discover truth. Kunda (1990) illustrated a host of domains where such motivated reasoning occurs. Strikingly, the vast majority of these domains concern self-relevant judgments—for example, people are inclined to seek, believe, and remember information that depicts themselves as smarter, healthier, and more socially desirable (Kunda, 1990; Mercier and Sperber, 2011). But judgments are typically defined as moral if they have “disinterested elicitors,” thus lacking immediate self-relevance (Haidt, 2003). Consequently, to evaluate whether *post hoc* reasoning drives moral judgments, we must consider cases in which the judgments have no direct self-relevance.

In such cases, people’s moral judgments can indeed influence subsequent reasoning processes in a motivated manner. When people see an issue in moral terms, they view tradeoffs about the issue as impermissible or taboo (Tetlock, 2003), and their judgments fall prey to various framing effects (Ritov and Baron, 1999; Sunstein, 2005; but see Connolly and Reb, 2003; Tanner and Medin, 2004). Moral judgments can also bias judgments of procedural justice, whereby people view judicial proceedings as more fair to the extent the outcomes are consistent with their own moral views (Skitka and Houston, 2001; Skitka, 2002). In general, these studies illustrate that motivated reasoning can work in the service of moral judgments, buttressing judgments that perceivers have already made. But the critical claim of Haidt’s model involves the process of arriving at moral judgments *themselves*.

Perhaps the most compelling method of evaluating Haidt’s claim that reasoning follows moral judgments is to jointly probe these judgments and the supporting reasons that people provide for them. Using this method, studies have shown that people sometimes judge behaviors wrong but seemingly cannot provide justificatory reasons, illustrating a phenomenon dubbed “moral dumbfounding” (Haidt et al., unpublished). Participants in Haidt et al.’s (unpublished) study read stories designed to depict disgusting yet harmless actions (e.g., consensual incest; eating a disease-free human cadaver), and although many people judged the actions to be wrong, they sometimes directly stated that they could not explain why. Haidt and Hersh (2001) reported similar results for harmless sexual behaviors (homosexual sex, unusual masturbation, and incest). Participants assigned a moderate amount of moral condemnation, and dumbfounding was observed among 49% of conservatives (and 33% of liberals).

Cushman et al. (2006) adopted a related approach by assessing people’s justifications for three principles—the *action*, *intention*, and *contact* principles^5^—that underlie their moral judgments. Participants had to justify patterns of judgments that conformed to the principles (e.g., that an action story was morally worse than an omission story), whereby sufficient justifications cited “a factual difference between the two cases and either claimed or implied that it was the basis of his or her judgments.” (Cushman et al., 2006, p. 1084). Any other justifications can be considered insufficient and are akin to instances of moral dumbfounding. Cushman et al. (2006) reported a sizeable proportion of dumbfounding for the intention principle (68%), but dumbfounding was less prevalent for the contact (40%) and action principles (19%).

##### Intuitive judgment

The second key claim of Haidt’s model is that intuitions or emotions directly influence moral judgments. Participants in Haidt et al. (1993) read stories describing harmless actions that were disgusting (e.g., having sex with a dead chicken, then cooking and eating it) or disrespectful (e.g., cleaning the bathroom with a cut up national flag), and their reported negative affect better predicted their moral judgments than did their judgments of harm. Similarly, Haidt and Hersh (2001) and Haidt et al. (unpublished) showed that wrongness judgments were better predicted by “gut feelings” or negative affect than by harm judgments.

Wheatley and Haidt (2005) hypnotized participants to feel disgusted by certain key words, then had them read moral violations, half of which contained the hypnotic disgust word. Participants rated the actions as more morally wrong when the hypnotic disgust word was present. Schnall et al. (2008) report similar findings: participants who were induced to feel disgusted (e.g., with a fart spray or disgusting film clip) made more severe moral judgments than control participants, although this was true only for people highly conscious of their own physical sensations. Eskine et al. (2011) found that people judged behaviors as more morally wrong after first drinking a bitter beverage, as opposed to a sweet or neutral one (but this pattern obtained only among conservative participants).

#### Limitations of Haidt’s Model

The evidence for Haidt’s model may not be widely generalizable to many types of moral violations or intuitions. Although Haidt’s definition of intuition appeals to a broad evaluative distinction between good and bad, most attempts to manipulate affective-based intuitions have focused on disgust specifically (Wheatley and Haidt, 2005; Schnall et al., 2008; Eskine et al., 2011). Similarly, most scenarios used to test Haidt’s model have involved disgust-based violations (e.g., Haidt et al., 1993; Haidt and Hersh, 2001; Haidt et al., unpublished). Widespread focus on disgust may overstate the role of intuition and the presence of dumbfounding. Disgust is elicited by the mere occurrence of a norm violation, whereas other moral emotions—such as anger—respond to the agent’s intentions (Russell and Giner-Sorolla, 2011a). People thus have difficulty justifying feelings of disgust but not feelings of anger (Russell and Giner-Sorolla, 2011b), suggesting that moral dumbfounding may be far less prevalent in non-purity domains. Indeed, when examining harmful behaviors, Cushman et al. (2006) observed dumbfounding in a majority of participants for just one of three moral judgment principles (the other dumbfounding rates were as low as 19%).

Even when focusing specifically on disgust, recent evidence provides a strong challenge to the claim that moral judgment is driven primarily by intuition or emotion. In a meta-analysis of over 50 studies, Landy and Goodwin (2015) report that the effect of induced disgust on moral judgment is verifiable but small (*d* = 0.11), and it disappears once correcting for publication bias. Furthermore, the paradigmatic cases of putatively harmless purity violations (e.g., Haidt et al., unpublished) are typically *not* perceived as harmless, which thereby explains people’s persistence in deeming them wrong (Gray et al., 2014; Royzman et al., 2015).

Lastly, studies of Haidt’s model typically ask participants whether behaviors are *wrong*, rather than *morally wrong* (Haidt et al., 1993; Haidt and Hersh, 2001; Schnall et al., 2008; Haidt et al., unpublished). These judgments, however, are distinct. Inbar et al. (2009) found that 45% of people said there was something “wrong with couples French kissing in public,” which likely reflects not judgments of *moral* wrongness, but rather judgments of social-conventional violation.^6^ Consistent with this suggestion that wrongness may not always have moral connotations, people are more willing to describe negative actions as “wrong” than as “morally wrong” (O’Hara et al., 2010). Importantly, this is not true for blame: people did not differentially describe negative actions as “blameworthy” versus “morally blameworthy” (O’Hara et al., 2010). Whereas blame has unambiguously moral connotations, wrongness does not.

### Greene: Dual Process Model of Moral Judgment

**FIGURE 5:**
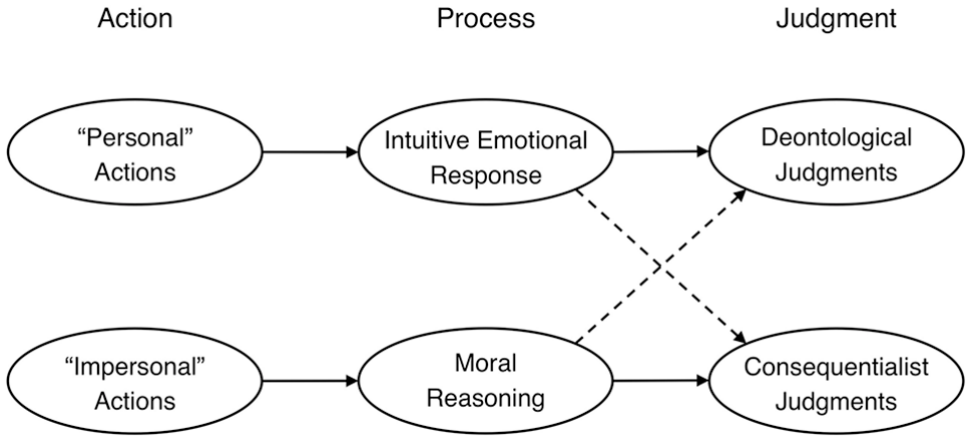
**Greene’s dual-process model of moral judgment**.

Greene’s (2007, 2013) model asserts that moral judgments are driven not just by intuitive/emotional processes but also by conscious reasoning processes. This dual process distinction has been proposed as a domain-general account of human cognition (Epstein, 1994; Sloman, 1996; Slovic et al., 2004; but see Keren and Schul, 2009). Critically, Greene’s (2007) model, shown in **Figure 5**, posits that these two processes underlie different types of moral judgment: “deontological judgments, judgments that are naturally regarded as reflecting concerns for rights and duties, are driven primarily by intuitive emotional responses,” whereas “consequentialist judgments, judgments aimed at promoting the greater good, are supported by controlled cognitive processes that look more like moral reasoning” (Paxton and Greene, 2010, p. 513).^7^

#### Evidence for Greene’s Dual-process Model

Greene’s model was inspired by a pair of moral dilemmas in which a runaway trolley is on course to kill five innocent workers. In the switch scenario, the hypothetical intervention is flipping a switch to divert the trolley onto a side track, killing a single worker tied to the tracks. In the footbridge scenario, the intervention is pushing a large man over a footbridge, stopping the trolley, and killing the man. Although both actions save five people and kill one, most people deem the switch intervention to be permissible and thus consistent with consequentialism but the footbridge intervention to be impermissible and thus inconsistent with consequentialism (Foot, 1967; Thomson, 1985; Petrinovich et al., 1993; Greene et al., 2001; Hauser et al., 2007). The explanation, according to Greene’s (2007, p. 43) model, is that “people tend toward consequentialism in the case in which the emotional response is low and tend toward deontology in the case in which the emotional response is high.”

Initial evidence for this model came from a seminal fMRI study by Greene et al. (2001) that compared “personal” dilemmas like *footbridge*, wherein the action involved direct bodily harm, to “impersonal” dilemmas like *switch*. Brain regions associated with emotional processing exhibited greater activation for personal than impersonal dilemmas, whereas regions associated with working memory showed greater activation for impersonal than personal dilemmas. People also took longer to judge personal actions appropriate than inappropriate, suggesting that it takes additional time to override the dominant emotionally aversive response.

If emotion underlies deontological judgments specifically, then counteracting people’s negative emotional responses should increase the acceptability of personal actions. Indeed, participants judged the *footbridge* action (but not the *switch* action) to be more appropriate after watching a funny video (Valdesolo and DeSteno, 2006). Patients with damage to the VMPFC, which is critical for healthy emotional functioning, have dulled physiological responses when considering harmful actions (Moretto et al., 2010) and are therefore more likely than controls to judge personal actions appropriate (Ciaramelli et al., 2007; Koenigs et al., 2007). In contrast, control participants show strong emotional aversion to engaging even in simulated harmful behavior, which predicts their rejection of hypothetical personal actions (Cushman et al., 2012).

If conscious reasoning underlies consequentialist judgments specifically, then taxing people’s cognitive processing capacities should impact these judgments. Consistent with this prediction, Greene et al. (2008) showed that whereas the frequency and speed of deontological judgments were unchanged by cognitive load, consequentialist judgments were slower with cognitive load than without. Relatedly, Conway and Gawronski (2013) found that cognitive load selectively weakened consequentialist (but not deontological) judgments. These findings are difficult to explain on Haidt’s model—if judgments are driven by immediate intuitive responses, then cognitive load should not affect the speed or content of these judgments. Moreover, participants in Greene et al.’s (2008) study made consequentialist judgments about personal actions 60% of the time, which also presents a challenge for Haidt’s model, as it suggests substantial deliberative reasoning despite the highly emotional content of these personal actions.

#### Limitations of Greene’s Model

Greene’s model may overstate the role of emotion in moral judgment by often probing first-person judgments (e.g., “Is it appropriate for you to…”), rather than third-person judgments. People respond more deontologically when considering their own actions (Nadelhoffer and Feltz, 2008). Thus, heightened emotional responses may be driven partially by the personal implications of the action (e.g., possible punishment, impression management), rather than purely by features of the action itself (cf. Mikhail, 2008). In fact, Borg et al. (2006) have noted that several brain regions implicated by Greene et al. (2001, 2004) are likewise involved in self-referential processing.

Further, the personal/impersonal distinction is coarse and perhaps inaccurate (Mikhail, 2007; McGuire et al., 2009), as it is not clear which features differentiate these categories, nor whether people consistently respond to them in the predicted fashion. McGuire et al. (2009) reanalyzed Greene et al.’s (2001) response time findings and showed that the differences were driven by a small subset of outlier personal dilemmas, which were uniformly (and quickly) judged inappropriate. Greene (2009) now agrees that the criteria distinguishing personal from impersonal actions are inadequate but notes that the veracity of the dual-process model does not depend on this. The model’s key claim (Greene, 2009) is that emotional and deliberative processes lead, respectively, to deontological and consequentialist judgments, however, these processes are elicited initially. This is true, but it seems to weaken Greene’s model, as it cannot predict the differential elicitation of these distinct processes.

A final challenge regards the utility of the distinction between deontological and consequentialist judgments. Recent evidence indicates that the supposedly consequentialist judgments revealed by classic moral dilemmas are more closely linked to egoist concerns than to concerns about the greater good (Kahane et al., 2015; see also Bartels and Pizarro, 2011). These findings add to a growing concern that moral dilemma scenarios may fail to adequately capture everyday moral judgment (Bloom, 2011; Bauman et al., 2014).

### Summary of Processing Models

Processing models seek to describe the psychological processes that give rise to moral judgments. Haidt argues that intuition alone drives most moral judgments, whereas Greene argues that both intuition and reasoning are critical. We can better understand these discrepant claims and findings by invoking the principles identified by information models. Studies of Haidt’s model primarily examine cases in which an agent acts intentionally, without apparent exculpatory justification; the key question for perceivers is therefore whether the act itself was negative. This norm-violation detection is often intuitive, and since the other information elements are held constant—causality and intentionality present, justification absent—no further information processing is required and moral judgments also appear intuitive. In contrast, studies of Greene’s model primarily examine cases in which an agent performs an intentional action that is indisputably negative, such as killing an innocent person; the key question for perceivers is therefore whether the action is justified by its positive consequences. These studies show that some actions are more easily justified (e.g., those not involving direct physical harm) and that reasoning often drives this process of considering justifications. Taken together, intuition is prominent when detecting initial norm violations, and conscious reasoning is prominent when weighing these early intuitive responses against potential countervailing considerations. As such, intuition and reasoning are both critical for moral judgment, but their relevance emerges in different ways and at different stages of the judgment process.

## Integration and Conclusion

This article has reviewed dominant models of moral judgment, organizing them in a theoretical framework of information processing that has been widely influential in models of cognitive psychology (Rosch, 1978; Marr, 1982) but neglected in models of morality. This framework aims to specify the information elements that shape moral judgments and the psychological processes that bring these judgments to bear. Information models address the first aim, identifying the particular information features that guide moral judgments (Shaver, 1985; Schlenker et al., 1994; Weiner, 1995; Cushman, 2008). These models examine a variety of moral judgments (e.g., blame, wrongness, responsibility) and emphasize different elements, but they coalesce around several key points. Central to moral judgments are variations in causality, intentionality, and mental states more generally, including beliefs, desires, and reasons or motives. Unintentional negative behaviors often receive substantial moral condemnation, particularly when they are preventable or controllable. Moral judgments are therefore guided by information about both the outcome and the mind.

A related set of models—biased information models—hold that the elements identified by information models are themselves guided by more basic moral judgments (Alicke, 2000; Knobe, 2010), such that negative events lead perceivers to make more culpable causal-mental judgments. Alicke offers a motivational explanation for this pattern: negative events trigger blame-validation, whereby perceivers inflate causal-mental judgments to justify initial negative feelings. Knobe offers a conceptual explanation: negative events fundamentally shape the way that people perceive mental states and causality. But these models face a central unresolved issue. Negative events, as instances of norm violations, often provide diagnostic evidence of an agent’s beliefs, motives, and intentions. Future research must therefore clarify whether such effects are attributable to morality per se or to concomitant informational elements.

Processing models specify the psychological processes that generate moral judgments, and existing models have primarily been interested in a dichotomy between intuition and reasoning (Haidt, 2001; Greene, 2007). These models endorse a central, and sometimes exclusive, role of intuition. To some degree, this should be unsurprising, as any judgment can be traced to a first principle that cannot be further justified. Just as people would have difficulty justifying their dislike of the color yellow (“it’s just ugly”), they will likewise have difficulty justifying why certain actions—such as committing incest or causing physical harm—constitute moral violations (“they’re just wrong”) (cf. Mallon and Nichols, 2011). Intuition is therefore prominent in detecting initial norm violations, or determining that *something* bad happened. Moral judgments themselves will also be intuitive when critical information elements concerning intentionality, justifications, and mental states are unambiguous and constant. In contrast, when these elements are equivocal or conflicting (e.g., when there is a potential justification for an initial negative event), moral judgments are additionally reliant on deliberate reasoning.

### An Information Processing Model of Moral Judgment

Moral judgments, like any others, fundamentally involve information processing, but existing models have typically examined either the information or the processing aspect of these judgments. A successful integrative model will be one that examines the relevant psychological processes as they relate not merely to eventual moral judgments themselves but to constitutive information elements. The subsequent sections examine how the insights of processing models apply to two distinct elements of information models—norm-violation detection and causal-mental analysis—and then discuss a recent a recent model, the Path Model of Blame (Malle et al., 2014), that adopts an integrative information processing approach.

#### Processes of Norm-violation Detection

For people to levy a moral judgment, they must first detect that a negative event has occurred—that some norm has been violated. Such norm-violation detection usually occurs quickly and triggers affective or evaluative responses (Ito et al., 1998; Van Berkum et al., 2009). Studies of Haidt’s model best exemplify the intuitive nature of this detection, showing that people easily, and sometimes without conscious justifications, classify particular behaviors as instances of moral violations (Haidt, 2001; Haidt and Hersh, 2001).

The critical findings of biased information models further support the intuitive basis of norm-violation detection. These models offer two key claims: first, that people identify negative events rapidly, in the form of spontaneous evaluations (Alicke, 2000) or initial moral judgments (Knobe, 2010); and second, that event negativity directly influences causal-mental judgments. Although the current analysis has challenged the second claim, the first claim is undisputed and strongly supported. This process of identifying an initial negative event or norm violation is also a stated or assumed aspect of information models (Shaver, 1985; Schlenker et al., 1994; Weiner, 1995; Cushman, 2008).

#### Processes of Causal and Mental Analysis

Identifying a negative event is only the first step en route to a moral judgment. It subsequently triggers an explanatory search for the causes of and reasons for the event (Malle and Knobe, 1997b; Wong and Weiner, 1981); and as several models have demonstrated, moral judgments are shaped by these causal-mental considerations (Cushman, 2008; Guglielmo et al., 2009; Gray et al., 2012), such as whether the event was intentional and what the agent’s more specific mental states were (beliefs, reasons, or motives).

The processes used to infer causality and mental states are varied and complex, including covariational and counterfactual reasoning, perspective taking, projection, and stereotyping (Hilton, 1990; Ames, 2004; Sloman et al., 2009; Waytz et al., 2010; Alicke et al., in press). These processes are triggered when the causal and mental features of the event at hand are (partially) ambiguous or conflicting, as in most naturalistic instances of moral judgment. In these cases, deliberative processes will often drive causal-mental analysis and, thus, moral judgments themselves. Studies of Greene’s model illustrate this pattern, showing that conscious reasoning substantially guides moral judgment when strong positive justifications conflict with highly negative norm violations. In contrast, when causal-mental features are unambiguous or non-conflicting, as in most studies of Haidt’s model, there is little need for deliberate reasoning; norm-violation detection and moral judgment become inseparable and largely intuitive.

Consequently, there is no compelling evidence that moral judgments are inherently either intuitive or deliberative. Which process dominates will depend on the nature and strength of the information regarding causality, intentionality, and mental states; but regardless of which process dominates, this causal-mental information is nonetheless considered (cf. Kruglanski and Gigerenzer, 2011). Ambiguous or conflicting information elicits deliberative processing, as when, for example, evaluating a genuine moral dilemma in which multiple courses of action involve different outcomes, tradeoffs, or motives for acting; unequivocal or non-conflicting information elicits intuitive processing, as when evaluating a single course of action for which someone’s motives are clearly specified or easily assumed (Monin et al., 2007). Researchers typically choose the strength and ambiguity of this information in service of particular theoretical perspectives. Subtle linguistic differences can help illustrate the point: “Smith was dead” leaves unspecified a perpetrator’s causal and intentional involvement (likely triggering more deliberate analysis of these features), whereas “Smith was murdered” obviates the need for deliberate analysis of causality and intentionality (although it may trigger analysis of the agent’s motives). Casting further doubt on attempts to characterize moral judgment as either intuitive or deliberative is the fact that even when judgments appear to be intuitive, this may actually reflect the automatization of prior conscious reasoning (Pizarro and Bloom, 2003; Mallon and Nichols, 2011).

#### The Path Model of Blame

**FIGURE 6:**
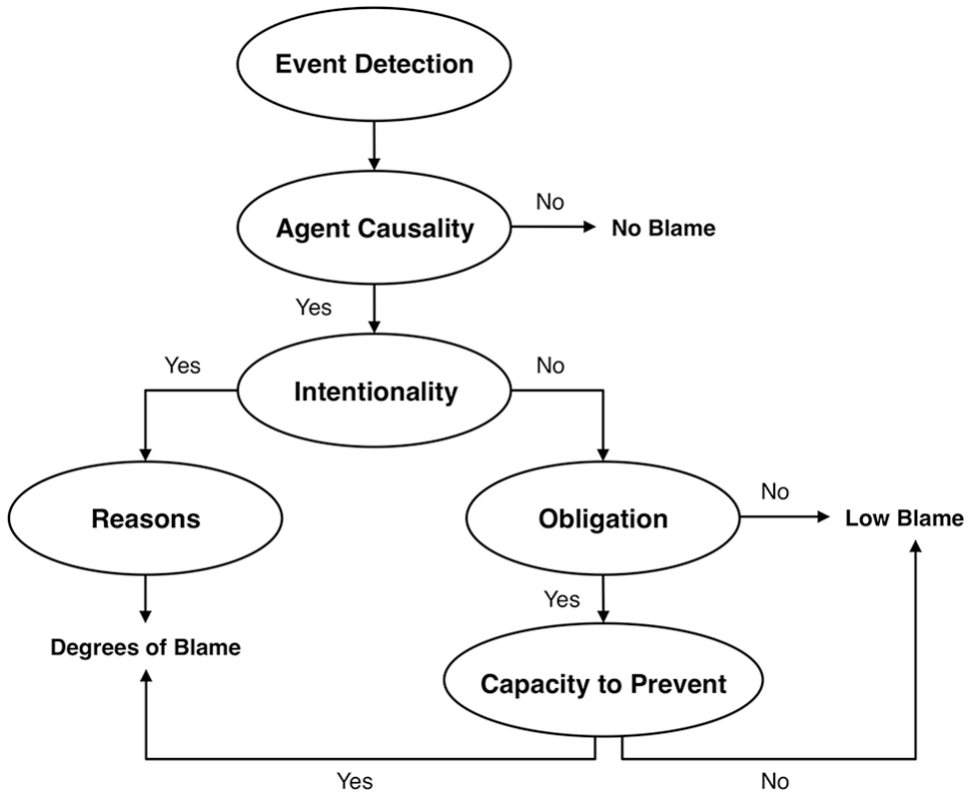
**Malle et al.’s Path Model of Blame**. Reprinted from Malle et al. (2014) with permission from Taylor and Francis Ltd.

A recent model, the Path Model of Blame (Malle et al., 2014; see **Figure 6**), adopts an explicit information processing view of moral judgment by considering the distinct processes of norm-violation detection and causal-mental analysis, and by specifying how information acquisition and integration underlie blame judgments. The model asserts that blame is initiated by the detection of a negative event or outcome (personal injury, environmental harm, and so on), which is typically an intuitive process. Perceivers then consider various information components en route to blame, but they do so in a particular processing order, which can manifest via either intuitive or deliberative processing. Perceivers assess the causality of the negative event in question and then, if it was agent-caused, they consider whether it was intentional. From there, blame unfolds via different paths: if the event is perceived to be intentional, perceivers consider the agent’s reasons or motives for acting; if perceived to be unintentional, perceivers consider the agent’s obligation and capacity to prevent the event.

The Path Model has notable similarities with several information models, particularly in recognizing the importance of the specific features of causality (Shaver, 1985; Weiner, 1995; Cushman, 2008), intentionality (Shaver, 1985; Cushman, 2008), reasons (Shaver, 1985), and preventability (Schlenker et al., 1994; Weiner, 1995). Like Cushman’s (2008) model, the Path Model also makes explicit that unintentional negative behavior can receive substantial blame. However, the Path Model extends previous models by specifying a processing hierarchy of information features, by identifying separate paths to blame depending on intentionality, and by clarifying how both intuitive and deliberative processes can shape blame. Recent evidence supports the information processing structure of the Path Model. In particular, when people find out about negative events and have an opportunity to acquire additional information, they do so in the order that the model posits, and this holds true even when they face strong time pressure and thus must rely on intuitive processing (Guglielmo and Malle, under review).

## The Future of Moral Psychology: Directions and Suggestions

Conceptualizing moral judgment in a framework of information processing facilitates a synthesis of previous research, helping to clarify the claims of existing models and illustrate their interconnections. Such a framework can likewise help guide future research, particularly by focusing on the affective basis of moral judgment, by diversifying the stimuli and methodologies used to study moral judgment, and by remaining grounded to the descriptive and functional questions of how and why our moral judgments operate as they do, rather than the normative questions of whether they operate correctly.

### Affect and Emotion

There is much debate concerning role of emotion in moral judgment. Researchers do not consistently disentangle intuitive judgment from emotion-influenced judgment; and though evidence for the former is relatively strong, evidence for the latter is weaker and has many possible theoretical interpretations (Chapman and Anderson, 2011; Pizarro et al., 2011; Landy and Goodwin, 2015). Emotionally arousing actions are often deemed permissible, and those lacking emotional salience are often judged immoral (Haidt et al., 1993; Greene, 2007; Koenigs et al., 2007). Moreover, even when considering highly emotional stimuli, greater deliberation (Pizarro et al., 2003a; Bartels, 2008) or weaker sensitivity to one’s bodily states (Schnall et al., 2008) considerably dulls the effects of emotion on moral judgments. Much additional research is needed—using a wider range of populations, stimulus items, and measures of emotion—before it becomes clear how, and to what extent, emotional mechanisms impact moral judgment (Huebner et al., 2009).

Importantly, any effect of emotion on moral judgment can arise only after causal and mental analysis (cf. Mikhail, 2007). If moral emotions stem from “negative feelings about the *actions or character* of others” (Haidt, 2003, p. 856, emphasis added), then they are predicated upon preceding causal-mental analysis. But negative *affect* may arise prior to such analysis, setting the process of moral judgment in motion. Negative events elicit rapid affective or evaluative responses (Ito et al., 1998; Van Berkum et al., 2009) and trigger processes of explanation and sense-making (Malle and Knobe, 1997b; Wong and Weiner, 1981). Thus, negative affect may lead perceivers to analyze agents’ causal and mental contribution, which thereby can elicit specific emotions such as anger (Russell and Giner-Sorolla, 2011a; Laurent et al., 2015c). In this way, negative affect motivates causal-mental analysis, rather than a search for blame-consistent information specifically. Knowing simply *that* a negative event has occurred is not enough for moral judgment (or moral emotion); people need to know *how* it occurred. And to make this determination, they appeal to the causal-mental structure of the event.

This conceptualization, whereby people interpret their negative affect within an explanatory framework prior to experiencing emotion, is consistent with cognitive appraisal theories of emotion (Barrett, 2006a; Barrett et al., 2007). On these accounts, “core affect” arises from the constant valuation of environmental stimuli (e.g., concerning harmfulness or helpfulness) and leads to emotion via the application of a conceptual framework that categorizes and explains the affect (Barrett, 2006a). In the context of moral judgment, causal-mental analysis provides the conceptual framework, appraising negative affect and thus giving rise to emotional experience and moral judgment.^8^

### Judgment Timing and Information Search

One domain in which the predictions from various models are decisively testable is that of timing. Many models assume, at least implicitly, that people make certain judgments before others. Both Cushman (2008) and Malle et al. (2014) posit that causality and mental state judgments precede blame. Knobe’s (2010) model predicts that initial moral judgments (e.g., about goodness or badness) precede mental state judgments, though the latter may precede full-fledged blame. Alicke’s (2000) model suggests that blame (in the form of spontaneous evaluations) should occur prior to judgments about causality and mental states. Testing these predictions about timing can further clarify the way in which moral judgments unfold and can adjudicate between claims made by existing models.

The claims of several models also have implications for perceivers’ search for information. Some models imply that, when assessing negative events, perceivers will try to actively acquire information about an agent’s causal involvement and mental states, as these most strongly guide blame (Cushman, 2008; Malle et al., 2014). Recent evidence supports such patterns of information seeking behavior (Guglielmo and Malle, under review). Alicke’s model, in contrast, might predict that sufficiently negative events will elicit blame and perceivers will rarely seek additional information about mental states (unless they have to justify their blame judgments). Processing models imply that when people are emotionally engaged, they may fail to notice or search for consequentialist information (e.g., how many people will be saved as a result of pushing the man off the footbridge).

### Domains, Contexts, and Measurement of Moral Judgment

In addition to attending to the integration of information and processing models, the study of morality will likewise benefit from further diversity and integration. Scholars have long focused on moral domains of harm and fairness, but Haidt (2007, 2008) and Graham et al. (2009, 2011) have emphasized the psychological relevance of various additional domains. Comparisons between moral domains are becoming more prevalent (Horberg et al., 2009; Young and Saxe, 2011; Chakroff and Young, 2015) and may soon yield conclusions about the extent to which existing models are widely, or narrowly, supported across domains.

Although moral judgments are typically studied intrapersonally—as cognitive judgments in the mind of a social perceiver—they undoubtedly serve important interpersonal functions (Haidt, 2001; McCullough et al., 2013; Malle et al., 2014). Moral judgments respond to the presence of social audiences (Kurzban et al., 2007), elicit social distancing from dissimilar others (Skitka et al., 2005), and trigger attempts to modify others’ future behavior (Cushman et al., 2009). Given that moral cognition ultimately serves a social regulatory function of guiding and coordinating social behavior (Cushman, 2013; Malle et al., 2014), further forging the connections between intrapersonal moral judgments and their interpersonal manifestations will be a critical direction for future research.

The measurement of moral judgment will also require detailed comparison and integration. Existing models primarily examine a single type of judgment—such as responsibility, wrongness, permissibility, or blame—and although all such judgments of course rely on information processing, they nonetheless differ in important ways (Cushman, 2008; O’Hara et al., 2010; Malle et al., 2014). Wrongness and permissibility judgments typically take intentional actions as their object of judgment (Cushman, 2008). Thus, judging that it is wrong (or impermissible) to X implies that it is wrong to *intentionally* X; it usually makes little sense to say that unintentionally X-ing is wrong. In contrast, responsibility and blame take both intentional and unintentional actions as their object of judgment. Thus, one can be judged responsible (Schlenker et al., 1994) or blameworthy (Cushman, 2008; Young and Saxe, 2009) even for purely unintentional negative behavior. Furthermore, because blame takes into account an agent’s reasons for acting, those who commit negative actions for justified reasons—such as self defense (Piazza et al., 2013)—can be deemed fully responsible yet minimally blameworthy (McGraw, 1987). Since these various moral judgments differ with respect to the amount and type of information they integrate, future work can further differentiate them by assessing both the temporal sequence of these judgments, and their sensitivity to different information features.

Finally, in reflecting the overwhelming preponderance of existing research, this review has focused on negative moral judgments. But what is the information processing structure of positive moral judgments? Relatively few studies have directly compared negative and positive moral judgments, although those that have done so reveal that these judgments are not mere opposites. Consistent with general negativity dominance effects (Baumeister et al., 2001; Rozin and Royzman, 2001), positive moral judgments are less severe than negative ones (Cushman et al., 2009; Goodwin and Darley, 2012), and certain categories of events—including outcomes that are unintended yet foreseen—elicit substantial blame when negative but essentially no praise when positive (Knobe, 2003a; Guglielmo and Malle, 2010). Since perceivers expect, by default, that others will try to foster positive outcomes and prevent negative ones (Pizarro et al., 2003b; Knobe, 2010), earning praise is more difficult than earning blame. Moreover, people often perceive that positive behavior is driven by ulterior motives (Tsang, 2006), which can quickly erode initial positive impressions (Marchand and Vonk, 2005). Thus, whereas positive and negative moral judgments share some information processing features—including sensitivity to intentionality and motives—the former are weaker and less broadly applicable.

### Beyond Bias

Claims of people’s deviation from normative or rational models of behavior abound in the psychological literature. As Krueger and Funder (2004) have shown, bias is often implied both by pattern X and by pattern not X, leaving it near impossible to discover unbiased behavior. As one example, viewing oneself more favorably than others constitutes a bias (self-enhancement), as does viewing oneself less favorably (self-effacement).

The emphasis on bias, and its supposed ubiquity, similarly exists in the moral judgment literature. Haidt (2001, p. 822) notes that “moral reasoning is not left free to search for truth but is likely to be hired out like a lawyer by various motives,” and many theorists appear to agree with this portrayal of biased judgment. The problem, however, is that opposing patterns of judgment are taken as evidence of such bias. The designation “outcome bias” implies that relying on outcome information connotes bias. To avoid biased judgment, perceivers should ignore outcomes and focus on the contents of the agent’s mind. In contrast, consequentialist accounts hold that “consequences are the *only* things that ultimately matter” (Greene, 2007, p. 37), which implies that perceivers should substantially—or even exclusively—rely on outcome information.

We have therefore doomed perceivers to be inescapably biased. Whatever judgments they make (e.g., whether using outcome information fully, partially, or not at all), they will violate certain normative standards of moral judgment. It is time, then, to move beyond charges of bias (cf. Bennis et al., 2010; Elqayam and Evans, 2011; Krueger and Funder, 2004). Future research will be more fruitful by focusing not on normative questions of how “good” or “correct” moral judgments are but on descriptive and functional questions: How do moral judgments work? And why do they work this way?

## Conclusion

This paper advanced an information-processing framework of morality, asserting that moral judgment is best understood by jointly examining the information elements and psychological processes that shape moral judgments. Dominant models were organized in this framework and evaluated on empirical and theoretical grounds. The paper highlighted distinct processes of norm-violation detection and causal-mental analysis, and discussed a recent model—the Path Model of Blame (Malle et al., 2014)—that examines these in an explicit information processing approach. Various suggestions for future research were discussed, including clarifying the roles of affect and emotion, diversifying the stimuli and methodologies used to assess moral judgment, distinguishing between various types of moral judgments, and emphasizing the functional (not normative) basis of morality. By remaining cognizant of the complex and systematic nature of moral judgment, exciting research on this topic will no doubt continue to flourish.

## Conflict of Interest Statement

The author declares that the research was conducted in the absence of any commercial or financial relationships that could be construed as a potential conflict of interest.

## Footnotes

1 According to Schlenker et al.’s (1994) model, intentionality is only incidentally relevant, representing one way in which events may be controllable.

2 “Bias” often carries normative connotations of error, but this is not the intended meaning here, since the models reviewed in this section disagree about whether their posited patterns reflect judgmental error. The current analysis invokes the more neutral meaning of “bias,” merely connoting a particular tendency.

3 Falsification is possible by challenging the temporal or causal relationship between variables, such as by showing that blame guides spontaneous evaluations (in which case the term *spontaneous* evaluations would be a misnomer). This strategy requires manipulation of variables, but blame and causal-mental judgments are measured variables by definition. Further, few studies examine the timing of these judgments, making it difficult to challenge temporal relationships between variables.

4 Robbennolt’s (2000) meta-analysis of outcome bias effects on blame also obtained an average effect size of *r* = 0.17. However, this represents the zero-order outcome bias effect (i.e., without controlling for any related inferences); the residual outcome → blame path would surely reveal a smaller effect size.

5 The action principle holds that harm caused by action is worse than harm caused by omission; the intention principle holds that intended harm is worse than harm brought about as a side-effect; the contact principle holds that harm caused by physical contact is worse than harm caused without physical contact.

6 Moreover, the question wording in this and other studies (“Is there anything wrong with…?”) sets a low threshold for assent and may thus elicit artificially high endorsement.

7 The model also posits that the emotion → consequentialism connection and the reasoning → deontology connection—depicted in **Figure 5** as dashed lines—are possible but rare.

8 Negative affect itself also requires appraisal—at minimum, that the event in question is negative.
